# Acoustic stimulation during slow wave sleep shows delayed effects on memory performance in older adults

**DOI:** 10.3389/frsle.2023.1294957

**Published:** 2024-01-05

**Authors:** Marina Wunderlin, Céline J. Zeller, Korian Wicki, Christoph Nissen, Marc A. Züst

**Affiliations:** ^1^University Hospital of Old Age Psychiatry and Psychotherapy, University of Bern, Bern, Switzerland; ^2^Graduate School for Health Sciences, University of Bern, Bern, Switzerland; ^3^Division of Psychiatric Specialties, Geneva University Hospitals (HUG), Geneva, Switzerland

**Keywords:** slow wave sleep, acoustic stimulation, old age, episodic memory, multiple stimulation nights

## Abstract

**Introduction:**

In young healthy adults, phase-locked acoustic stimulation (PLAS) during slow wave sleep (SWS) can boost over-night episodic memory consolidation. In older adults, evidence is scarce and available results are inconsistent, pointing toward reduced PLAS-effectiveness. We argue that multiple stimulation nights are required for effects to unfold in older individuals to compensate for age-related reductions in both SWS and memory performance. We test this assumption in a longitudinal within-subject design.

**Methods:**

In a larger previous project, older adults participated in a three-night intervention receiving either real-PLAS (STIM group) or sham-PLAS (SHAM group). Encoding and immediate recall of face-occupation pairs was administered on the evening of the first intervention night (session one), with feedback-based retrievals ensuing on all following mornings and evenings across the intervention. To test for the benefit of the real-PLAS over sham-PLAS intervention within participants, 16 older adults [age_mean_: 68.9 (SD: 3.7)] were re-invited receiving the real-PLAS intervention exclusively. This resulted in a SHAMSTIM group (*n* = 9; T1: sham-PLAS intervention, T2: real-PLAS intervention) and a STIMSTIM group (*n* = 7; T1 and T2: real-PLAS intervention).

**Results:**

While the STIMSTIM group exhibited highly similar responses during T1 and T2, the SHAMSTIM group exhibited a significantly higher increase in memory performance at T2 (real-PLAS) compared to T1 (sham-PLAS). These gains can be attributed to the late stages of the experiment, after three nights of real-PLAS, and remained stable when correcting for changes in baseline sleep quality (PSQI) and baseline cognitive ability (Montreal Cognitive Assessment) between T1 and T2.

**Conclusions:**

We show that in older adults, PLAS-induced memory effects are delayed and manifest over the course of a three-night-PLAS intervention. Our results might explain the lack of effects in previous PLAS studies, where memory performance was solely assessed after a single night of PLAS.

## 1 Introduction

Phase-locked acoustic stimulation (PLAS) during slow wave sleep (SWS) is a method that has sparked considerable interest due to its potential to increase memory performance. PLAS algorithms can read the signal from an electroencephalogram (EEG) of sleeping participants in real-time, detect when the brain has shifted into SWS and eventually apply short, auditory stimuli in phase with the ongoing slow wave (SW) activity (Ngo et al., 2013). The method takes advantage of SWS representing a critical stage for episodic memory consolidation (Diekelmann and Born, 2010; Rasch and Born, 2013). In the EEG, SWS is represented by high-amplitude, slow (<4 Hz) waves with SW-peaks and troughs representing phases of depolarization and hyperpolarization, respectively (Steriade et al., 1993). Although we refer to the most widely used definition of the slow-wave frequency range (0.5–4 Hz; Fehér et al., 2021), we do not exclude the possibility that some slow waves might exhibit a broader frequency range. The neocortical SW peaks coordinate the occurrence of thalamo-cortical sleep spindles, oscillatory events of 12–16 Hz (Rasch and Born, 2013). This temporal coupling of SW-peaks and spindles further determines activity in the hippocampus (Rasch and Born, 2013), a critical structure for memory consolidation (Squire, 2004). The goal of PLAS is to enhance SW amplitudes, SW occurrence, SW/spindle synchronization, and—as a downstream effect—promote more efficient memory consolidation. While the exact mechanisms how acoustic stimuli enhance temporo-cortical synchronization are not yet clear, our meta-analysis showed that PLAS is able to boost both SWS and memory performance in healthy young adults (Wunderlin et al., 2021).

Episodic memory performance decreases with age (Nyberg et al., 1996). Likewise, the quality of SWS (= SWS duration, amplitude and density of slow waves), sleep spindles and their temporal co-occurrence decline with increasing age (De Gennaro and Ferrara, 2003; Mander et al., 2017; Helfrich et al., 2018; Züst et al., 2023). Hence, since PLAS can increase both SW-spindle synchrony as well as episodic memory performance, it would in theory be an ideal intervention tool in older age. Unfortunately, evidence from studies applying PLAS in older adults is scarce and currently available results are inconsistent with regard to efficacy (Wunderlin et al., 2021). One study showed a positive effect of PLAS on overnight memory retention compared to a night without PLAS (Papalambros et al., 2017) while another study found no such benefit (Schneider et al., 2020). We previously argued that certain characteristics of PLAS study designs should be altered to render them better suited and therefore more effective in older adults (Wunderlin et al., 2020). Most importantly, due to the concurrent decline in memory performance and sleep quality observed in older adults, we posit that a single night of PLAS and a single memory assessment might be insufficient for effects to manifest.

In a larger previous project (Wunderlin et al., in press), we introduced a study design where older adults were either allocated to an intervention group receiving three consecutive nights of real-PLAS (intervention (STIM-) group) or a control group receiving three nights of sham-PLAS (control (SHAM-) group). Memory performance (relative to a pre-experimental baseline) was assessed on each morning and each evening across the experimental nights. While the focus of the previous study is to investigate between subject differences in memory performance (intervention vs. control group), here, we investigate within-subject differences of the real-PLAS intervention vs. the sham-PLAS intervention in a longitudinal design. For this purpose, participants who were in the SHAM group at T1 (= the previous study) were re-invited to undergo the real-PLAS intervention (T2; SHAMSTIM group). Additionally, participants from the original STIM group (T1) were re-invited to undergo the real-PLAS intervention for a second time (T2) to serve as a control group and to assess long-term stability of the real-PLAS intervention (STIMSTIM group). Our main hypothesis was that memory benefits might not yet be observed after a single night of real-PLAS but might only unfold in a delayed manner across the intervention.

## 2 Materials and methods

### 2.1 Experimental design and procedures

**Figure 1:**
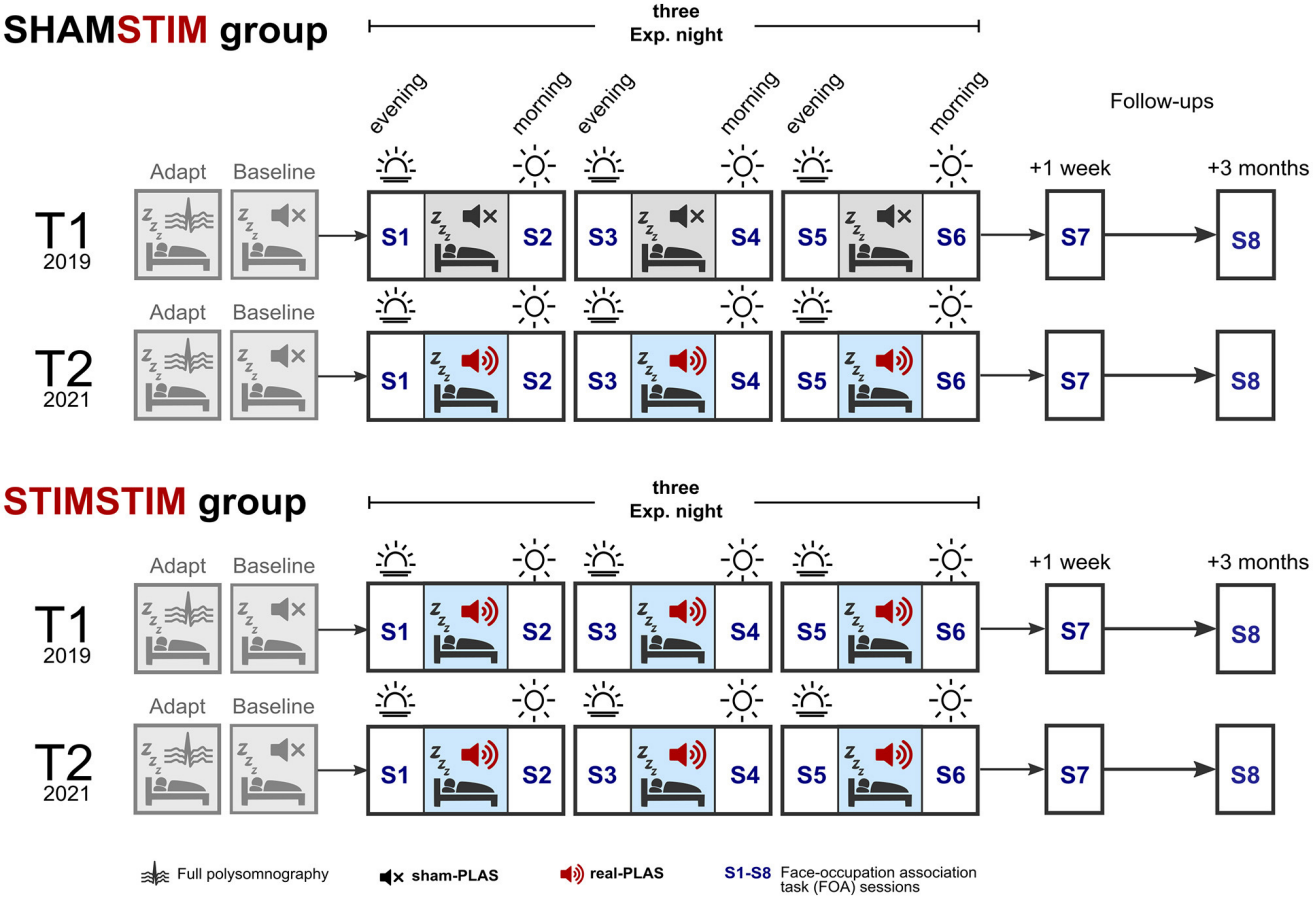
Study design. Participants spent five nights in the sleep laboratory, the first being an adaptation night and the second a baseline night, where sham-PLAS was administered (depicted by a black mute sign). At T1 (top row in both groups, occurring in 2019), the SHAMSTIM group received three consecutive nights of sham-PLAS whereas the STIMSTIM group received three nights of real-PLAS (depicted by red speakers). Participants returned to the sleep laboratory at T2 (in 2021), receiving real-PLAS exclusively (bottom rows in both groups). Retrieval of face occupation associations (FOA) was tested across the three nights on each morning (S2, S4, and S6) and evening (S3 and S5). Initial encoding and an immediate recall took place on the evening prior to the first experimental night (S1). Memory performance was reassessed at a 1-week and 3-months follow up.

The data presented here stems from a larger previous project (Wunderlin et al., in press) where—in a between subject's design—older adults underwent either a three-night real-PLAS intervention (STIM group) or a three-night sham-PLAS intervention (SHAM group). We refer to this larger previous project as “T1.” To assess differences between the real-PLAS and the sham-PLAS protocol within study participants, rather than between participants, we re-invited participants from the initial SHAM group (T1) to undergo the real-PLAS protocol in this new study arm, which we refer to as “T2.” The group who had received sham-PLAS under T1 and now real-PLAS under T2 is referred to as the SHAMSTIM group (see Figure 1). Participants from the original STIM group (T1) were also re-invited to undergo the real-PLAS intervention a second time (T2). We refer to this group as the STIMSTIM group, which served two purposes: (1) These participants allow for an investigation of long-term stability of the PLAS- intervention, (2) these participants constitute a control group, where no differences between T1 and T2 were expected.

Prior to the three experimental nights (E1, E2, and E3), both protocols included a preceding baseline night, where sham-PLAS was administered as well as an adaptation/screening night where potential sleep pathologies were determined (see Figure 1). Under sham-PLAS, the stimulation algorithm detected SWs in the online EEG signal of sleeping participants and set time markers for stimulations without transmitting a sound. Acoustic stimuli were transmitted under the real-PLAS condition exclusively. Episodic memory performance was assessed by a face-occupation association (FOA) task, where 40 face stimuli were randomly paired with 20 occupations. The first session contained the initial encoding and immediate recall and occurred on the evening of the first experimental night (S1, baseline memory assessment). Feedback-based retrieval sessions were assessed across the three experimental nights on mornings (S2, S4) and evenings (S3, S5). On the morning of the last experimental night (S6, post-intervention) the retrieval session contained no feedback. Memory performance was re-assessed at a 1-week (S7) and a 3-month (S8) follow up. The task was designed to produce a gradual increase in proficiency after repeated learning. The objective was to test the influence of repeated PLAS interventions on cumulative learning. See Figure 1 for an overview of the study design. During both T1 and T2, participants adhered to their individualized sleep schedules, maintaining a consistent duration in bed from lights off to lights on throughout the intervention. Subjective sleep quality was measured across the nights through sleep diaries (Carney et al., 2012) and the Stanford Sleepiness Scale (Hoddes et al., 1973). T1 and T2 were ~1–2 years apart. This study was approved by the cantonal ethics committee of Bern (KEK Bern) and informed consent was obtained from all participants.

### 2.2 Sample

In the original previous study (Wunderlin et al., in press) at T1, participants were quasi-randomly allocated to the real-PLAS or the sham-PLAS condition using a covariate-adaptive randomization technique to ensure comparable basic cognitive levels, ages, and self-reported sleep quality levels. From the original previous study (T1), 16 participants agreed to re-enroll in the new study arm (T2), nine of which were originally in the sham-PLAS condition and seven in the real-PLAS condition. The SHAMSTIM group consisted of nine participants [age T2: 71.3 (2.45), seven females, two males] and the STIMSTIM group of seven participants [age T2: 68.4 (4.35), six females, one male]. At both T1 and T2, exclusion criteria were impaired hearing, sleep disorders, irregular sleep patterns, pre-existing neurological or psychiatric conditions, intake of psychotropic drugs and non-fluency in German. All participants were screened for sleep apnea and restless leg syndrome at both T1 and T2. Subjective sleep quality and health was assessed via the Pittsburgh Sleep Quality Index (PSQI, Buysse et al., 1989). General cognitive ability was measured via the Montreal Cognitive Assessment (MoCA, Nasreddine et al., 2005). Table 1 displays changes in Moca-Score, PSQI, and age from T1 to T2.

**Table 1:** Means and standard errors for the time passed, sleep quality, cognitive ability, and age per group and time point.

	**SHAMSTIM group**			**STIMSTIM group**		
	**T1**	**T2**	**p**	**T1**	**T2**	**p**
Δ time (days)	462 (226)		**-**	403 (206)		-
Age	70.2 (3.07)	71.3 (2.45)	**-**	67.3 (4.27)	68.4 (4.35)	-
MOCA	28.1 (1.17)	26.7 (1.87)	**0.031**	27 (1.29)	28.4 (1.4)	0.118
PSQI	4.67 (2)	5.11 (1.54)	0.225	3.29 (1.5)	3.14 (1.07)	0.846

Significant p-values (p <0.05, bold) depict differences between T1 and T2. Δ time = time difference (in days) between T1 and T2. MOCA = Montreal Cognitive Assessment, maximum score: 30, >26: cognitively healthy. PSQI = Pittsburgh Sleep Quality Index: <5: normal sleep.

### 2.3 Memory task

The Face-Occupation Association (FOA) task was programmed using Presentation^®^ software (Version 20.2, Neurobehavioral Systems, Inc., Berkeley, CA, www.neurobs.com). At each time point (T1/T2), a unique set of 40 face stimuli was used. The face stimuli per set were chosen based on similar questionnaire ratings for perceived age, income, attractiveness and recognizability of 120 faces from a face image database (Karras et al., 2018). Both sets were gender balanced. For each participant, the set of faces was randomly paired with one of 20 occupations, each occupation being represented by a male and a female face. Ten occupations displayed a cognitive/academic focus (such as architect) and the other 10 a manual/physical focus (such as gardener).

Encoding of FOA pairs took place in two runs on the evening of the first experimental night (S1, Figure 1). In both runs, the pairs were displayed in a random sequence where the face stimuli were positioned to the left of a fixation cross and the corresponding occupation to the right. The stimuli remained visible for 5,000 ms, followed by an inter-stimulus interval (ISI) of 500 ms. In the cued recall phase, participants were presented with the faces and tasked with verbally recalling the associated occupations. Their responses were documented and transcribed after the session.

### 2.4 EEG system and acoustic stimulation

We used a 128-channel MicroCel Geodesic Sensor net (400 series Geodesic EEG System^TM^) along with a Physio16 input box (both Magstim EGI, Eugene, OR, USA) for EEG and polysomnographic measurements. The initial sampling rate was 500 Hz, referenced to Cz. For the analyses, the data was down-sampled to 200 Hz and preprocessed using the PREP pipeline (Bigdely-Shamlo et al., 2015). An experienced and certified rater conducted polysomnographic assessment of sleep stages (W, N1, N2, N3, REM) in accordance with the guidelines established by the American Academy of Sleep Medicine (Iber, 2007). The PLAS algorithm is described elsewhere in more detail (Ruch et al., 2022; Wunderlin et al., 2022). In short, the algorithm bases the detection of SW peaks on the ideal topographic representation of a SW peak. This renders its performance largely amplitude-independent, which increases detection performance in older adults, where SW amplitudes are becoming progressively lower compared to younger adults (Wunderlin et al., 2022). Acoustic stimuli were transmitted via sleepphones^®^ (AcousticSheep LLC). Each participant's individual hearing threshold was assessed by a hearing test. The individual threshold (plus a fixed digital stereo-mixer amplitude of 0.5) served as the target stimulus intensity. The acoustic stimuli contained 50 ms of pink noise and the mean volume of presented stimuli during the experimental nights was 68.8 dB(A) [CI: 68 – 69.9 dB(A)]. Based on previous work in the field, the stimulation target was the rising phase of the positive half wave (Ferster et al., 2019). The mean phases of stimulation were −31.5° (8) in the STIMSTIM group at T1 and −32.7° (8.4) at T2. In the SHAMSTIM group, the mean phases were −23° (14.6) for T1 and −25.7° (13.5) for T2. As 0° and −180° represent the peak and preceding trough of a SW, respectively, our analyses confirmed that the stimulations occur at the target (rising) phase of the slow wave.

### 2.5 Statistical analysis

Analyses were performed in R (R Core Team, 2021) version 4.1.1. and MATLAB version R2019a (The MathWorks, Natick, Massachusetts) using the toolboxes EEGLAB (Delorme and Makeig, 2004) and FieldTrip (Oostenveld et al., 2010).

To investigate physiological responses to PLAS, grand mean event related potentials (ERPs) as well as grand mean event related spectral perturbations (ERSPs) were calculated per night (BL, E1–E3), per time point (T1, T2) and per group (SHAMSTIM, STIMSTIM). The continuous data was epoched between −1.5 and 3 s for ERPs and between −4.5 and 5.5 s for ERSPs, time-locked to the real-PLAS and sham-PLAS stimulations. For ERPs, the whole window was used as a baseline correction. For ERSPs, Morlet wavelet transforms were calculated for a frequency range from 0.5 to 30 Hz (resolution of 0.25 Hz) and a baseline correction was applied using the time window from 2 to 2.5 s. Within each group, the ERPs and ERSPs of all four nights were compared between T1 and T2 using non-parametric permutation tests as implemented in FieldTrip. In short, paired *t*-tests were calculated for each channel-time(-frequency)-pair between the conditions of interest, grouping samples with *p* < 0.001 into clusters, and testing the cluster's summed *t*-value against randomly shuffled permutations (*n* = 1,000, mixing conditions), recomputing the test statistic for each of these permutations. The cluster-level test is performed by comparing the sizes of empirical clusters to those obtained from permutations (*p* < 0.05). Positive clusters indicate an increase in activity between the two time points and negative clusters indicate decreases in activity. In the ERSP analysis, significant positive and negative clusters were visualized by aggregating data across all channels in the time-frequency domain. The level of electrode involvement was represented on a color scale ranging from 0 to +/- 1, denoting the range of zero to 128 electrodes engaged within the specific positive or negative cluster. This was executed independently for each experimental night, but an average was computed to emphasize clusters consistently present across all nights. While the cluster-based tests compared all corresponding nights between T1 and T2, we further investigated T1/T2 differences in relative power increases from the baseline to the experimental nights. For this, median power values at temporospatial-frequency clusters of interest were first extracted from all four nights. The frequency bins and time windows of interest were chosen based on results of the previous cluster-analysis: 0.75–1.5 Hz (SO), 1–4 Hz (delta), 12–16 Hz (spindles) and 4–8 Hz (theta), post stimulus window (0–2 s), induced trough (0.25–0.75 s), induced peak (1–1.5 s). Frontal channels were used as electrodes of interest, except for spindles were centro-parietal electrodes were used based on previous reports of spindle topography (Rasch and Born, 2013). Next, we computed a mean over the tree experimental nights for each cluster and calculated a difference score by subtracting the respective cluster's value from the baseline night (E-BL). Difference scores were compared between T1 and T2 using paired *t*-tests or Wilcoxon signed rank test in case of non-normal distributions as determined by Shapiro-Wilk tests.

Whole night spectral power was obtained by first cutting the continuous EEG data to N2/N3 sleep stages only. Next, Fast Fourier transformations were performed on continuous 5-second segments with 50% overlap using Hanning tapers. The average power for frequency bins up to 50 Hz was calculated for each electrode. Frequency ranges of interest were 0.6–1.6 Hz (SO), 1–4 Hz (delta), 12–16 Hz (spindles), and 4–8 Hz (theta). Log-transformed mean power values over frontal channels (and over central channels for spindle power) were extracted per subject, night and time point. Differences between experimental nights and the baseline night as well as differences between the respective nights at T1 vs. T2 were calculated within each group using paired *t*-tests or Wilcoxon signed rank tests (depending on normality of the distribution).

To investigate differences in relative memory performance between T1 and T2, we first compared the slopes for the performance increment from the baseline assessment (session one) to post intervention (session six) between T1 and T2 within both the SHAMSTIM and the STIMSTIM group using paired *t*-tests. Relative memory performance incorporating all recall sessions two to eight was investigated using a binomial probit-linked (generalized) linear mixed-effects model (GLMM). The bobyqa optimizer was applied with the iteration limit set to 100,000. Memory performance was predicted on a single-trial level (“memory”) for each group (SHAMSTIM and STIMSTIM) separately. The variables *participant ID* (9 in the SHAMSTIM group/7 in the STIMSTIM group) and *face stimulus* (80) were entered as random effects. GLMM 1 was calculated as follows: “memory ~ session^*^time + (1 | participant ID) + (1 | face stimulus),” including the interaction between sessions and time. Note that the factors session and time are coded with the first session (pre-intervention, baseline) and the first time point (T1) as reference. Effects can therefore be interpreted as contrasts against the pre-intervention session at T2 compared to T1. Additional models including potential covariates were calculated. Models without covariates were compared to models with covariates using Likelihood-ratio χ^2^-tests.

To assess whether PLAS-induced enhancement in the SO and spindle band was associated with the relative memory increase at post-intervention and the two follow-ups, individual physiological responsiveness values were calculated per participant. For the 16 participants receiving real-PLAS under T2, we extracted median power values in all four nights for the SO (0.75–1.5 Hz) and the spindle band (12–16 Hz) at time windows suggested by the previously computed cluster-based ERSPs-analysis (SO: 0–2 s; Spindle: 1–1.5 s). For each experimental night, we subtracted the baseline night's values to determine PLAS-induced increases in the three experimental nights. Finally, to obtain a single value per participant, we calculated a weighted mean over the three nights, using the number of applied stimulations per night as a weight—accounting for inter-night variability of stimulation success. To test for associations between physiological response values and memory performance, we used regression analyses.

## 3 Results

### 3.1 Real-PLAS entrains slow waves and increases stimulation-locked slow-oscillatory, delta, theta, and spindle power

**Figure 2:**
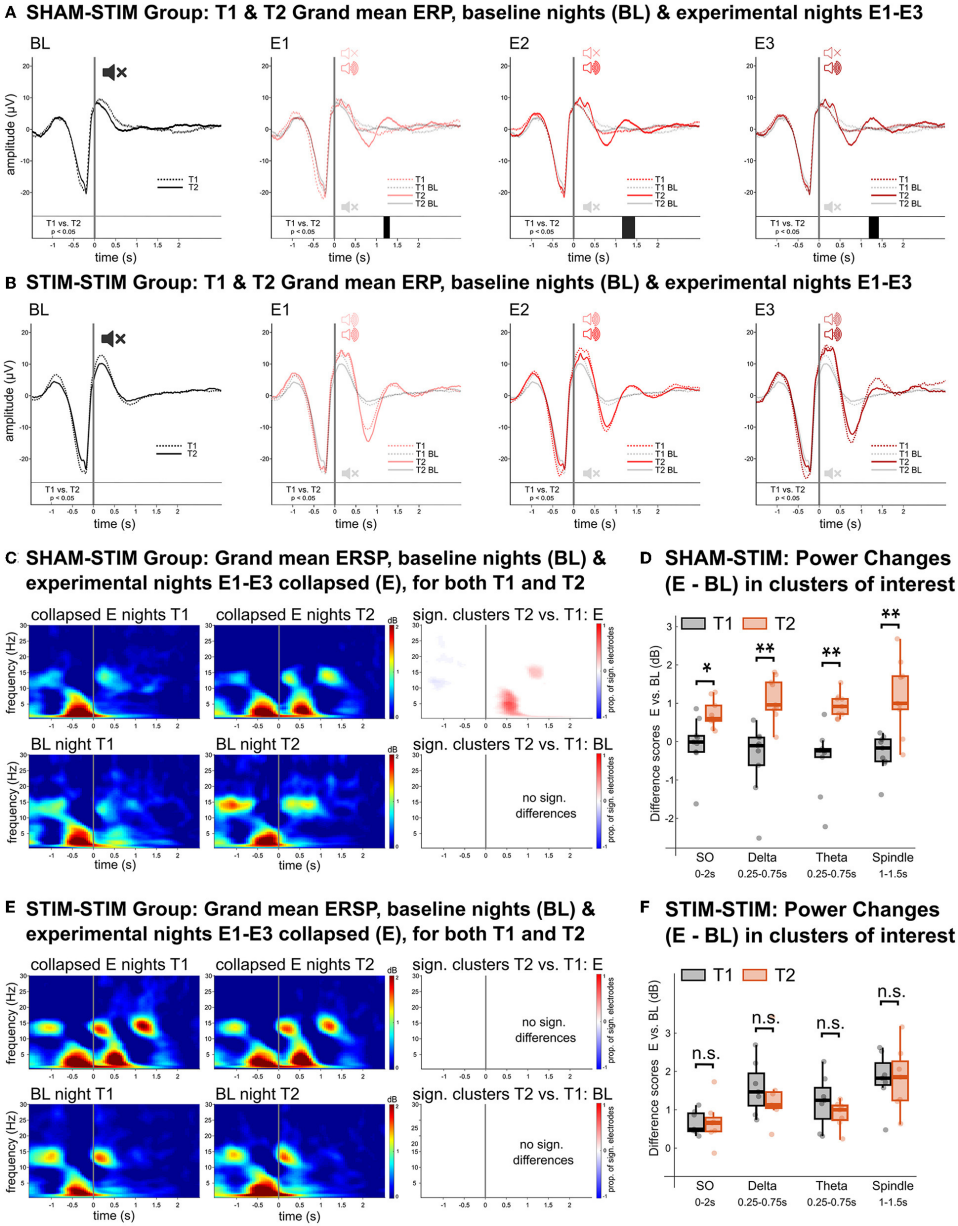
Physiological effects of real- vs. sham-PLAS. **(A, B)** Grand mean ERPs in channel Fz for the SHAMSTIM group **(A)** and the STIMSTIM group **(B)** for the baseline night (BL, black lines) and the three experimental nights (E1, E2, and E3, red lines). Solid lines represent the ERPs at T2 and dashed lines represent ERPs at T1. Directly above the x-axis are black bars representing time windows of significant differences between T1 and T2. The only differences between T1 and T2 are seen in the SHAMSTIM group, which received real-PLAS at T2 and sham-PLAS at T1 whereas there were no differences in the STIMSTIM group, which received real-PLAS during both time points. For within-intervention reference, the BL ERPs are embedded within the E1-E3 graphs. **(C, E)** Grand mean ERSPs over frontal channels for the SHAMSTIM and the STIMSTIM group, respectively. The top rows represent spectral activity during the collapsed experimental nights (E) for T1 **(left)** and T2 **(right)**. The bottom rows show the same difference for the baseline night. The graphs on the right display significant clusters (red = increased power, blue = decreased power) showing differences between T1 and T2. The proportion of involved electrodes in the cluster is represented by the color gradient, where 1 = 128 electrodes, positive effect (T2 > T1) and −1 = 128 electrodes, negative effect (T1 > T2). The only significant differences are seen in the SHAMSTIM group's experimental nights, where there was an increase in the SO (~0.75–1.5 Hz), delta (1–4 Hz), theta (4–8 Hz), and spindle (12–16 Hz) power corresponding to the entrained SW-trough and SW-peak under the real-PLAS condition (T2). **(D, F)** Difference scores between experimental nights and the baseline night for extracted median power values in time-channel-frequency-clusters of interest for T1 (black) and T2 (red), displayed for the SHAMSTIM group **(D)** and the STIMSTIM group **(F)**. The x-axis displays the frequency bins as well as the time-window of extraction, both of which were determined by the significant cluster in **(C)**. *p < 0.05, **p < 0.01.

To analyze the physiological response to PLAS, we calculated event related potentials (ERPs). Figure 2A displays significant differences between the corresponding nights at T1 and T2 for the SHAMSTIM group. In the three experimental nights (E1–E3), the real-PLAS intervention at T2 (red solid lines) induced an entrainment of SWs—reflected by an induced SW trough (0.25–1 s) and second SW peak (1–1.5 s)—when compared to the three experimental nights E1–E3 at T1, where sham-PLAS was administered (red dashed lines). Cluster-based statistical testing revealed statistically different time windows (*p* < 0.05) between T1 and T2 as marked by black bars above the x-axes. There were no differences between the two time points in the baseline night (BL) where sham-PLAS was employed for both T1 and T2. For within-intervention reference, the BL ERPs [dashed black line (T1), solid black line (T2)] are additionally plotted with the E1–E3 ERPs. Figure 2B displays T1 and T2 ERPs for the STIMSTIM group, which received real PLAS-stimulation during E1–E3 and sham-PLAS during the BL night at both time points. As expected, there were no significant differences between the T1 and T2 ERPs.

We next investigated differences in event related spectral perturbations (ERSPs) which display changes in spectral power within the stimulation window. For this analysis, we used the averaged signal from the three experimental nights (E). In the SHAMSTIM group (Figure 2C), cluster-based tests revealed an increase in power from T1 (upper panel, left) to T2 (upper panel, middle) in the SO (~0.75–1.5 Hz), delta (1–4 Hz), and theta (4–8 Hz) range corresponding to the induced SW trough as well as an increase in spindle (12–16 Hz) and SO power corresponding to the induced second SW peak [*p* < 0.05, see red (positive) clusters in Figure 2C, upper panel, right]. Such differences were not observed between the BL nights (Figure 2C, lower panel). Figure 2D displays difference scores (E – BL) in median power values at time-frequency-clusters of interest for both T1 (sham-PLAS) and T2 (real-PLAS). The power increase from the BL night to the E nights in the SO band post stimulus (0–2 s) was significantly higher at T2 compared to T1 (*p* < 0.0*5*). Furthermore, during the induced SW trough (~0.25–0.75 s) delta and theta power increases were significantly larger at T2 compared to T1 (*p* < 0.01). During the induced SW peak (~1–1.5 s), increases in spindle power were significantly different between T1 and T2 (*p* < 0.01). Figures 2E, F show that there was no significant power difference between T1 and T2 in the STIMSTIM group which received real-PLAS stimulation in all experimental nights. These results demonstrate that PLAS consistently induced an entrainment of SWs with stimulation-locked increases in SO-, delta, theta, and spindle power.

There were no systematic differences between T1 and T2 in global sleep parameters, such as sleep architecture or all-night spectral power. Of note, in the SHAMSTIM group, all-night spindle power was significantly higher in the third experimental night compared to the baseline night (V = 3, *p* = 0.02) at T2 but not at T1. However, this was neither observed in the other experimental nights, nor in the STIMSTIM group at any time point.

### 3.2 Real-PLAS induced increases in memory performance in a delayed manner

**Figure 3:**
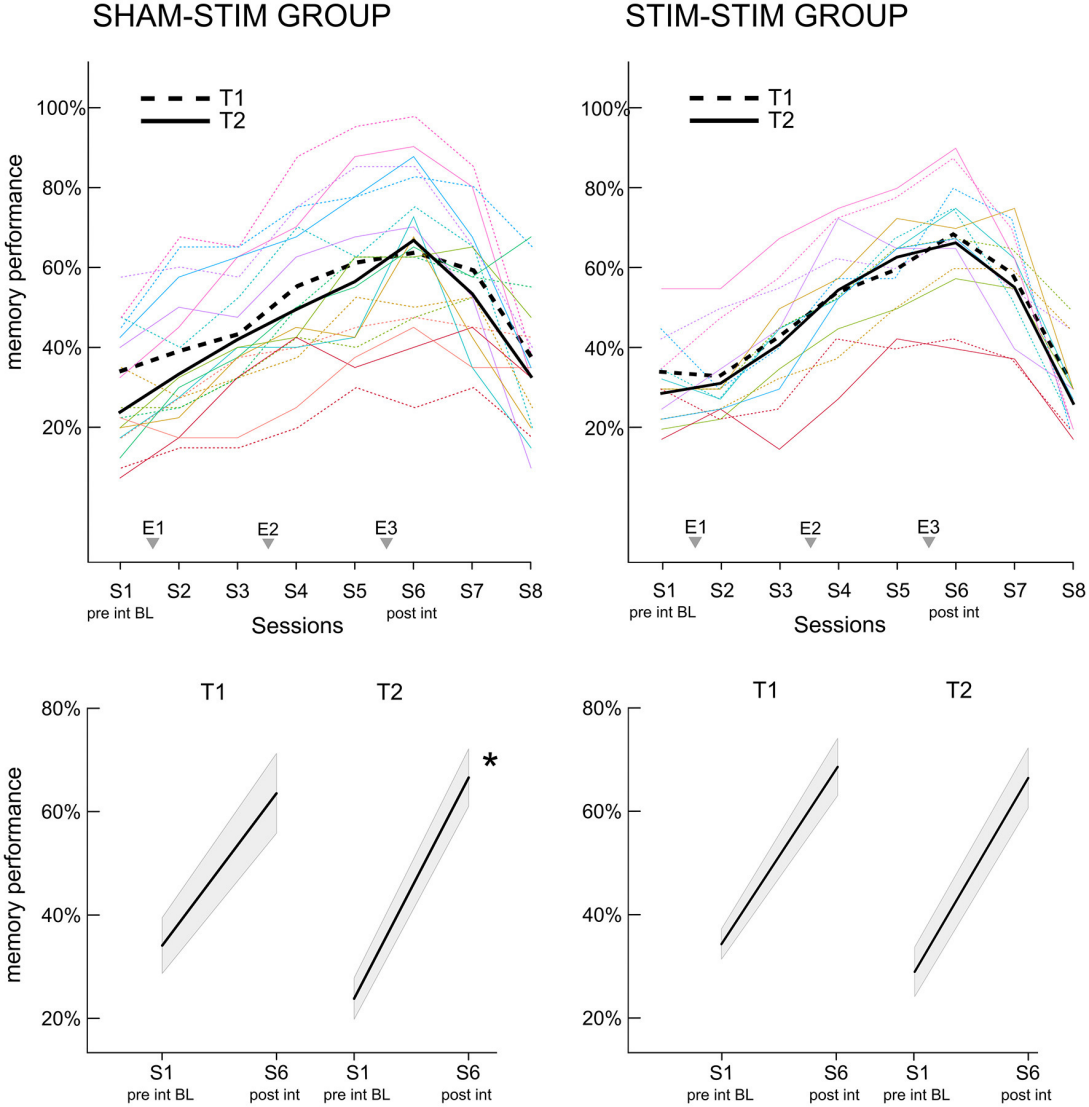
Increase in memory performance for the face-occupation association (FOA) task. **(Upper panel)** The x-axis displays the recall sessions for the FOA task. Recall took place at the baseline before the first experimental night (S1), at all morning sessions (S2, S4, and S6), all evening sessions (S3 and S5) as well as the two follow up sessions (S7: 1 week post intervention, S8: 3 months post intervention). The three experimental nights (E1–E3) are added to the x-axis for reference. Dashed lines represent the first time point T1, where the SHAMSTIM group **(left)** received sham-PLAS and the STIMSTIM group **(right)** received real-PLAS. Solid lines show the learning curves at T2, where both groups received real-PLAS. **(Bottom panel)** the slopes for the increase in memory performance from pre to post-intervention are depicted for both time points (T1, T2) and both groups [**(left)** SHAMSTIM group and **(right)** STIMSTIM group]. *p < 0.05.

To assess whether the increase in memory performance across the intervention differed within participants between the two time points, we analyzed the per-session and per-time-point percentage of correctly recalled face-occupation pairs. Figure 3 displays the learning curves for both T1 and T2, separately for the SHAMSTIM (left) and the STIMSTIM group (right). In the SHAMSTIM group but not the STIMSTIM group, the slope from the pre intervention baseline (S1, evening before the first experimental night) to post intervention (S6, morning after the third experimental night) was significantly steeper at T2 (0.42) compared to T1 (0.29, *t* = 3.2, *p* = 0.012, Hedges' *g* = 1.07). To incorporate all session scores on a single trial level in relation to the baseline (S1), while controlling for the varying difficulty of single trials as well as per-subject baseline differences, we calculated a GLMM with the face stimuli and the intercept as random factors. Table 2 displays the results of model 1 (GLMM 1 with the interaction term time x session, no covariates) for the SHAMSTIM (top, 5,760 observations) and the STIMSTIM group (bottom, 4,480 observations). In the SHAMSTIM group, there was a significant interaction for sessions three (*p* = 0.026, odds ratio = 1.39, evening after the first experimental night) and session six (*p* = 0.001, odds ratio = 1.63, post intervention) indicating an increase in relative memory performance under real-PLAS (T2) compared to sham-PLAS (T1) for the two sessions. There were no significant effects in the STIMSTIM group.

**Table 2:** Results of generalized linear mixed model (GLMM) 1 testing interactions between increases in memory performance at recall sessions two to eight (in relation to session one, baseline) and the time point T2 (in relation to T1).

	**Estimate**	**Std. error**	***Z***	***P***
**SHAMSTIM group: fixed effects**				
(Intercept)	−0.470	0.170	−2.757	0.006
Time T2	−0.373	0.148	−2.518	0.012
Session 2	0.156	0.101	1.536	0.125
Session 3	0.279	0.101	2.771	0.006
Session 4	0.627	0.101	6.200	<0.001
Session 5	0.805	0.102	7.894	<0.001
Session 6	0.883	0.102	8.619	<0.001
Session 7	0.740	0.101	7.296	<0.001
Session 8	0.101	0.101	1.006	0.315
Time T2: Session 2	0.175	0.148	1.185	0.236
Time T2: Session 3	0.326	0.146	2.227	**0.026**
Time T2: Session 4	0.209	0.146	1.427	0.154
Time T2: Session 5	0.241	0.147	1.632	0.103
Time T2: Session 6	0.486	0.149	3.263	**0.001**
Time T2: Session 7	0.207	0.147	1.409	0.159
Time T2: Session 8	0.219	0.147	1.495	0.135
**STIMSTIM group: fixed effects**				
(Intercept)	−0.439	0.144	−3.035	0.002
Time T2	−0.192	0.154	−1.248	0.212
Session 2	−0.046	0.114	−0.407	0.684
Session 3	0.240	0.112	2.139	0.032
Session 4	0.552	0.112	4.934	<0.001
Session 5	0.716	0.113	6.346	<0.001
Session 6	0.993	0.116	8.591	<0.001
Session 7	0.694	0.113	6.157	<0.001
Session 8	−0.076	0.114	−0.669	0.503
Time T2: Session 2	0.127	0.164	0.777	0.437
Time T2: Session 3	0.132	0.161	0.819	0.413
Time T2: Session 4	0.210	0.161	1.304	0.192
Time T2: Session 5	0.288	0.162	1.773	0.076
Time T2: Session 6	0.130	0.165	0.787	0.431
Time T2: Session 7	0.084	0.161	0.519	0.604
Time T2: Session 8	0.002	0.164	0.009	0.992

The top table displays results for the SHAMSTIM group which received real-PLAS during T2 and sham-PLAS during T1. The bottom table displays results from the STIMSTIM group, receiving real-PLAS during both T1 and T2. Subject and Face stimuli are in the model as random factors. P-values of significant interaction effects (p <0.05) are shown in bold.

However, in the SHAMSTIM group, the baseline performance significantly differed between T1 and T2 (T1: 0.34 vs. T2: 0.23, *t* = 2.96, *p* = 0.018). Hence, we calculated a GLMM 2 incorporating only the baseline performance and adding covariates potentially contributing to the drop in baseline performance. The time passed between T1 and T2, the change in general cognitive ability (MOCA-score) as well as the change in general sleep quality (PSQI-score) were added to model 2 as covariates. Model 2 revealed that the drop in baseline performance at T2 was significantly explained by a reduction in sleep quality (interaction Δ PSQI x time, *p* = 0.044). Hence, we re-calculated model 1 incorporating the drop in sleep quality as a covariate (model 3, GLMM 3). There was still a significant interaction between time and sessions three and six and model comparisons revealed that model 3 did not explain the data significantly better than model 1 (*p* > 0.6). Finally, to further ensure that the difference in baseline performance did not carry the significant time x session interactions, we calculated model 4 where memory performance was investigated in relation to session two (instead of session one). Session two was chosen here as the performance did not significantly differ between time points (T1: 39.2%, T2: 33.3%, *p* = 0.1). Model 4 (GLMM 4) revealed that the interaction time x session six was still significant (*p* = 0.031, odds ratio = 1.37), but not the interaction time x session three (*p* = 0.3). All models were repeated for the STIMSTIM group where no significant interactions were found. Together, these results indicate that real-PLAS at T2 accelerated the increase in memory performance (in relation to the baseline memory assessment) compared to sham-PLAS at T1 whereas there were no differences for participants that received real-PLAS at both time points. This memory benefit is not apparent immediately after the first PLAS-night (where most previous PLAS studies assessed memory performance), but rather during later stages of the intervention.

Finally, due to the functional relevance of slow wave activity and sleep spindles in memory performance, we investigated, whether PLAS-induced increases in the SO and spindle band (see Figures 2C, D) could predict relative increases in memory performance. Neither relative increases in SO nor spindle power significantly predicted relative memory increases at post-intervention or the two follow-up sessions (all *p* > 0.6). Note that the observed lack of effect may be attributed to the relatively small sample size, a situation which is not optimal for correlative analyses.

## 4 Discussion

In this longitudinal within-subjects study in older adults, we found that a three-night real-PLAS intervention entrained SW-activity and enhanced SO, spindle, delta and theta power time- locked to the entrained SW peak/trough. Importantly, we showed that within-subjects, a real-PLAS intervention compared to a sham-PLAS intervention increased episodic memory performance in a delayed manner—manifesting over the course of a three-night intervention. Lastly, this study demonstrated that physiological effects of real-PLAS were highly stable across time.

In older adults, evidence pointing toward PLAS-effects on memory performance is scarce and inconsistent (Wunderlin et al., 2020, 2021). We argued that due to decreases in SWS and sleep spindle quality (Mander et al., 2017; Helfrich et al., 2018), as well as declines in episodic memory functioning (Nyberg et al., 1996), older adults might require multiple PLAS nights for downstream effects on memory to unfold. Indeed, our results showed that memory effects were not yet evident on the morning after the first PLAS night. Furthermore, the only morning memory assessment showing a significant performance increase was the post-intervention session after three consecutive nights, providing support for the idea of a cumulative benefit over multiple nights. Our results could potentially explain the lack of memory effects found in previous PLAS studies, in which memory performance was exclusively evaluated in the morning after an isolated night of PLAS (Schneider et al., 2020). An isolated night of PLAS might simply not have wielded sufficient influence to substantially impact memory performance.

An alternative explanation as to why memory effects were delayed might be rooted in the possibility that PLAS could have impacted more than mere over-night memory consolidation. In our main GLM model (GLMM 1), we found that initial PLAS-induced benefits on memory performance occurred at session three, which is the memory assessment in the evening after the first PLAS-night. In our study, recall sessions were feedback-based which enabled additional encoding of face-occupation pairs at each session. Hence, PLAS might have not only positively impacted overnight memory consolidation, but also new learning upon waking or even over-the-day wake-dependent consolidation. This could explain why memory effects were not seen directly upon waking, but only later in the evening. Evidence in favor of this explanation stems from a study using an alternative stimulation approach to enhance SWS, namely transcranial electrical stimulation (tES). The authors showed that tES boosted SWS and was able to increase the capacity for new learning upon waking (Antonenko et al., 2013). However, the initial benefit on memory performance at session three was no longer seen at sessions four and five. While this could be accounted for by a potential initial stagnation for performance optimization, the effects in session three might have also been caused by a biased baseline memory assessment. All memory increases were investigated in relation to a pre-intervention baseline assessment. Critically, in the SHAMSTIM group, the baseline memory performance was significantly lower at T2 compared to T1. Therefore, the increase observed at session three might be an artifact of the lowered baseline performance, merely mirroring greater potential for performance increase when starting at a lower baseline. Hence, an additionally calculated GLM model (GLMM 4) investigated the robustness of memory effects, when using a baseline performance that is comparable between T1 and T2. In this model the effect in session three vanished, but the effect at post-intervention remained stable. However, it is possible that the observed post-intervention memory benefit could have depended on a synergy between the multi-night application and the extended PLAS-impact on other wake-dependent memory functions.

### 4.1 Limitations

Because the current study was based on a subsample of a larger previous project, balancing of study conditions was not possible: In the SHAMSTIM group, participants were always in the sham-PLAS condition first (at T1) and returned to the lab for the real-PLAS condition (T2). Such a design can introduce bias and confounding variables. Furthermore, the time passed between T1 and T2 was not balanced. However, both time points involved a baseline assessment of sleep with sham-PLAS, a baseline evaluation of memory performance as well as measurements of other variables that could potentially have an impact. We included potential confounding variables (such as change in cognitive functioning, change in sleep quality, the time passed between time points) as covariates in our statistical models and the observed effects remained stable.

Although PLAS led to an increase in episodic memory performance at post-intervention, this memory benefit was no longer apparent at a 1-week or 3-month follow-up session. Hence, memory effects from a three-night PLAS intervention do not seem to be long-lasting. It is possible that for long-term memory benefits, even more nights of PLAS might be needed. Critically, our results showed that PLAS' physiological effects were stable within a three-night intervention without displaying habituation effects. Furthermore, in the STIMSTIM group, PLAS physiological effects remained stable even in participants for whom more than 400 days passed in between the real-PLAS interventions. Other studies using multiple nights of PLAS further confirmed a lack of physiological habituation, rendering PLAS a good candidate for long-term applications (Debellemaniere et al., 2018; Lustenberger et al., 2022). With current developments in the field of PLAS-capable home-use devices (Zeller et al., 2023), future research can delve into options for long-term PLAS-applications and investigate whether memory increases can be sustainable.

## 5 Conclusions

In older adults, multi-night PLAS might be necessary to compensate for age-related decreases in both slow wave activity and memory performance. While most PLAS studies focus on over-night memory consolidation, it might be prudent to tap PLAS' full potential and make use of multiple PLAS-optimizable processes, such as over-night memory consolidation and post-sleep memory encoding. We provide evidence that PLAS increases learning rates for episodic memory in older adults, which renders it a promising tool to build upon for developing long-term interventions in memory-related conditions, such as mild cognitive impairment (MCI) or dementia.

## Data availability statement

The raw data supporting the conclusions of this article will be made available by the authors, without undue reservation.

## Ethics statement

The studies involving humans were approved by Cantonal Ethics Committee of Bern, Bern, Switzerland. The studies were conducted in accordance with the local legislation and institutional requirements. The participants provided their written informed consent to participate in this study.

## Author contributions

MW: Conceptualization, Data curation, Formal analysis, Funding acquisition, Investigation, Methodology, Visualization, Writing – original draft, Writing – review & editing. CZ: Investigation, Writing – review & editing. KW: Writing – review & editing. CN: Conceptualization, Writing – review & editing. MZ: Conceptualization, Data curation, Formal analysis, Funding acquisition, Investigation, Methodology, Supervision, Writing – review & editing, Project administration.
